# Crystal structure and Hirshfeld surface analysis of ethyl 2-{4-[(3-methyl-2-oxo-1,2-di­hydro­quinoxalin-1-yl)meth­yl]-1*H*-1,2,3-triazol-1-yl}acetate

**DOI:** 10.1107/S2056989018014561

**Published:** 2018-10-23

**Authors:** Nadeem Abad, Youssef Ramli, Tuncer Hökelek, Nada Kheira Sebbar, Joel T. Mague, El Mokhtar Essassi

**Affiliations:** a Laboratoire de Chimie Organique Hétérocyclique URAC 21, Pôle de Compétence Pharmacochimie, Av. Ibn Battouta, BP 1014, Faculté des Sciences, Université Mohammed V, Rabat, Morocco; b Laboratory of Medicinal Chemistry, Faculty of Medicine and Pharmacy, Mohammed V University, Rabat, Morocco; c Department of Physics, Hacettepe University, 06800 Beytepe, Ankara, Turkey; d Laboratoire de Chimie Bioorganique Appliquée, Faculté des Sciences, Université Ibn Zohr, Agadir, Morocco; e Department of Chemistry, Tulane University, New Orleans, LA 70118, USA

**Keywords:** crystal structure, di­hydro­quinoxaline, hydrogen bond, π-stacking, Hirshfeld surface

## Abstract

The mol­ecule of the title compound, C_16_H_17_N_5_O_3_, is build up from two fused six-membered rings linked to a 1,2,3-triazole ring, which is attached to an ethyl azido-acetate group. The di­hydro­qinoxalinone portion is planar to within 0.0512 (12) Å and is oriented at a dihedral angle of 87.83 (5)° with respect to the pendant triazole ring. In the crystal, a combination of inter­molecular C—H⋯O and C—H⋯N hydrogen bonds together with slipped π-stacking [centroid–centroid distance = 3.7772 (12) Å] and C—H⋯π (ring) inter­actions lead to the formation of chains extending along the *c*-axis direction. Additional C—H⋯O hydrogen bonds link these chains into layers parallel to the *bc* plane and the layers are tied together by complementary π-stacking [centroid–centroid distance = 3.5444 (12) Å] inter­actions. The Hirshfeld surface analysis of the crystal structure indicates that the most important contributions for the crystal packing are from H⋯H (44.5%), H⋯O/O⋯H (18.8%), H⋯N/N⋯H (17.0%) and H⋯C/C⋯H (10.4%) inter­actions.

## Abstract 2

The di­hydro­qinoxalinone portion of the mol­ecule is planar to within 0.0512 (12) Å. In the crystal, a combination of C—H⋯O and C—H⋯N hydrogen bonds together with slipped π-stacking and C—H⋯π(ring) inter­actions lead to the formation of chains extending along the *c*-axis direction. The chains are linked into layers parallel to the *bc* plane by sets of four C—H⋯O hydrogen bonds and the layers are tied together by complementary π-stacking inter­actions.

## Chemical context   

**Figure 1:**
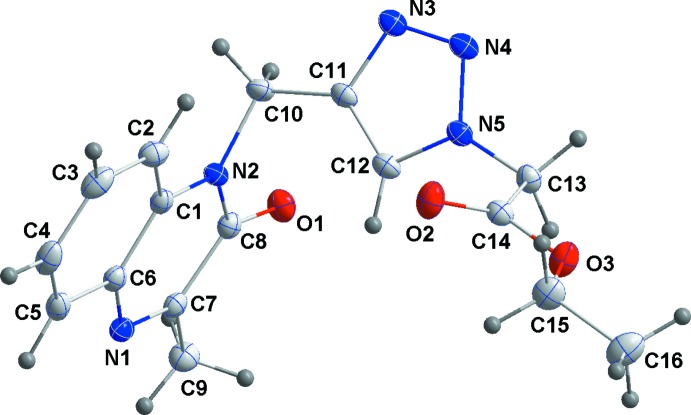
The title mol­ecule with the labelling scheme and 50% probability ellipsoids.

Quinoxaline derivatives, especially quinoxalinone, are of great importance in medicinal chemistry (Ramli & Essassi, 2015 ▸; Ramli *et al.*, 2017 ▸) and can be used for the synthesis of numerous heterocyclic compounds with various biological activities such as anti­bacterial (Griffith *et al.*, 1992 ▸), HIV (Loriga *et al.*, 1997 ▸), anti­microbial (Badran *et al.*, 2003 ▸), anti-inflammatory (Wagle *et al.*, 2008 ▸), anti­protozoal (Hui *et al.*, 2006 ▸), and anti­cancer (Carta *et al.*, 2006 ▸). In a continuation of our research work devoted to the study of cyclo­addition reactions involving quinoxaline derivatives (Ramli *et al.*, 2011 ▸, 2013 ▸; Abad *et al.*, 2018 ▸; Sebbar *et al.*, 2016 ▸), we report in this work the synthesis, using 3-methyl-1-(prop-2-yn­yl)-3,4-di­hydro­quinoxalin-2(1*H*)-one as dipolarophile and ethyl azido acetate as 1,3-dipole, and crystal structure of ethyl 2-{4-[(3-methyl-2-oxo-1,2-di­hydro­quinoxalin-1-yl)meth­yl]-1*H*-1,2,3-triazol-1-yl}acetate, C_16_H_17_N_5_O_3_ (Fig. 1 ▸).

## Structural commentary   

The mol­ecule of the title compound is build up from two fused six-membered rings linked to a 1,2,3-triazole ring which is attached to ethyl azido­acetate group (Fig. 1 ▸) (Sebbar *et al.*, 2014 ▸; Ellouz *et al.*, 2015 ▸).
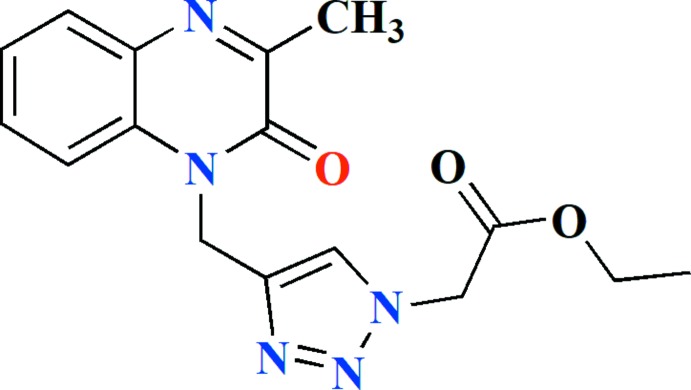


Atoms C8 and N2 are displaced from the mean plane through the di­hydro­quinoxalinone unit by 0.0367 (13) and −0.0512 (12) Å, respectively, with the remaining atoms within 0.0222 (15) Å of the plane (r.m.s deviation of the fitted atoms is 0.0234 Å). The pendant triazole ring is inclined to this plane by 87.83 (5)°.

## Supra­molecular features   

**Figure 2:**
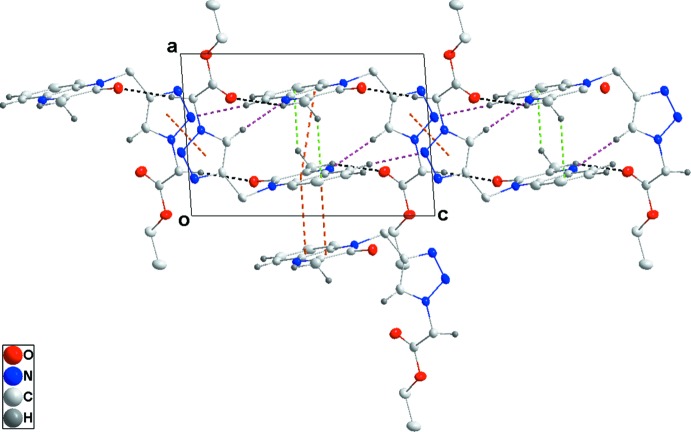
Detail of the inter­molecular inter­actions viewed along the *b*-axis direction. C—H⋯O and N—H⋯O hydrogen bonds are shown, respectively, by black and purple dashed lines. Slipped π-stacking and C—H⋯π (ring) inter­actions are shown, respectively, by orange and green dashed lines.

**Figure 3:**
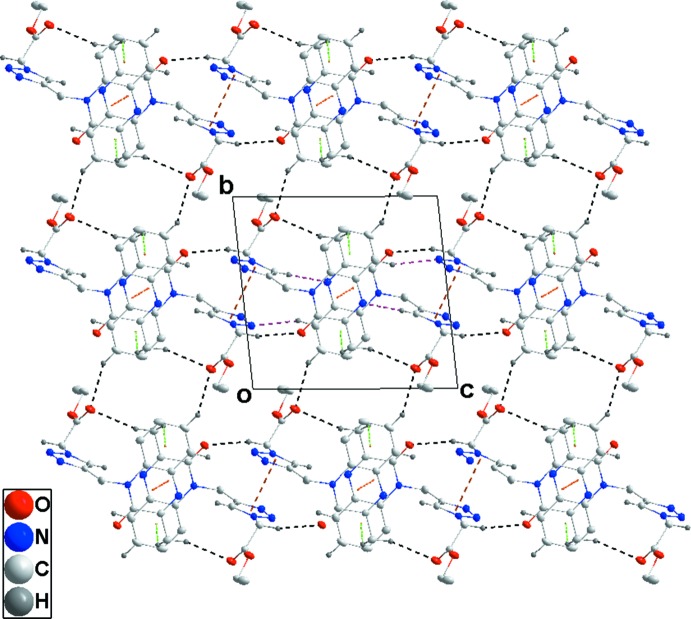
Plane view of one layer along the *a*-axis direction with inter­molecular inter­actions depicted as in Fig. 2 ▸.

Hydrogen bonding and van der Waals contacts are the dominant inter­actions in the crystal packing. In the crystal, C—H_Dhyqnx_⋯O_Ethazac_, C—H_Ethazac_⋯O_Dhyqnx_, C5—H_Dhyqnx_⋯N_Ethazac_ and C—H_Trz_⋯N_Dhyqnx_ (Dhyqnx = di­hydro­quinoxalin, Ethazac = ethyl azido­acetate and Trz = triazol) hydrogen bonds (Table 1 ▸) form chains extending along the *c*-axis direction (Figs. 2 ▸ and 3 ▸). These are reinforced by slipped π-stacking inter­actions between inversion-related *A* (N1/N2/C1/C6–C8) rings [centroid–centroid distance = 3.7772 (12) Å] and by complementary C—H_Dhyqnx_⋯*Cg*3 inter­actions [*Cg*3 is the centroid of the benzene ring *B* (C1–C6)] (Table 1 ▸ and Fig. 2 ▸). The chains are linked into layers parallel to the *bc* plane by sets of four C—H_Dhyqnx_⋯O_Ethazac_ hydrogen bonds (Table 1 ▸ and Fig. 3 ▸) with the layers linked along the *a*-axis direction by inversion-related slipped π-stacking inter­actions between the *A* and *B* rings [centroid–centroid distance = 3.5444 (12) Å] (Fig. 2 ▸).

## Hirshfeld surface analysis   

**Figure 4:**
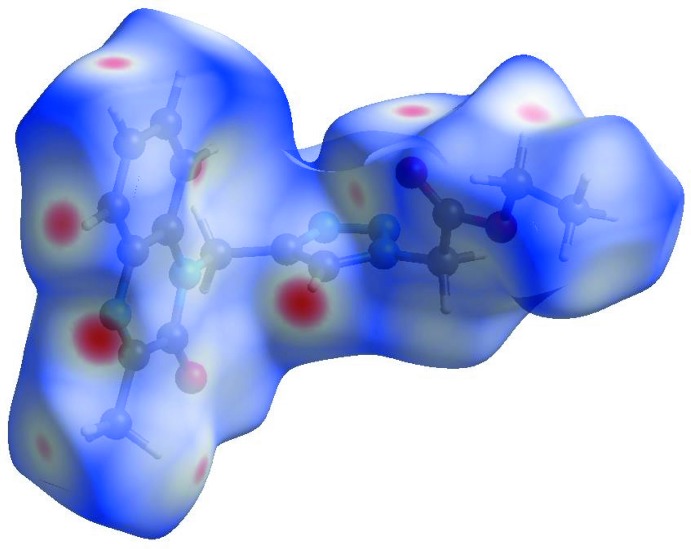
View of the three-dimensional Hirshfeld surface of the title compound plotted over *d*_norm_ in the range −0.2685 to 1.3470 a.u.

**Figure 5:**
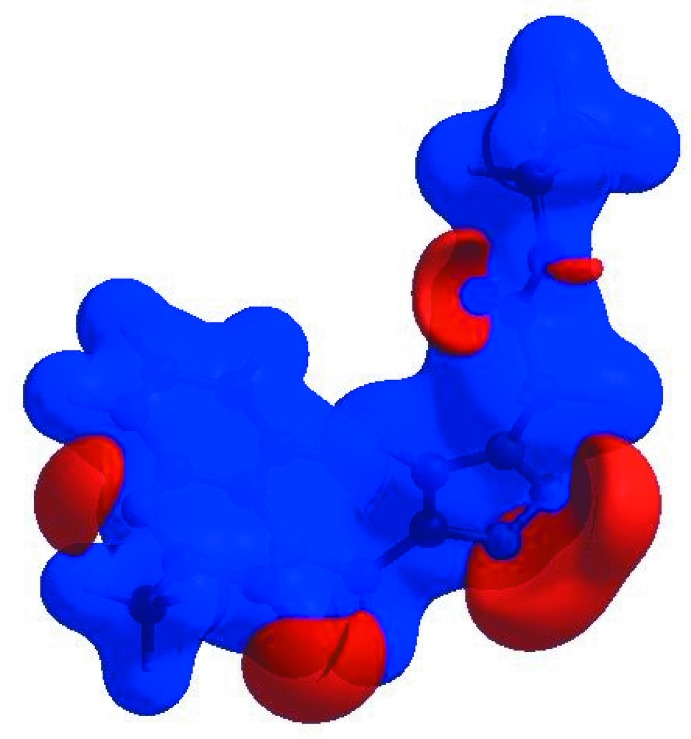
View of the three-dimensional Hirshfeld surface of the title compound plotted over electrostatic potential energy in the range −0.0500 to 0.0500 a.u. using the STO-3 G basis set at the Hartree–Fock level of theory. Hydrogen-bond donors and acceptors are shown as blue and red regions around the atoms, corresponding to positive and negative potentials, respectively.

Visualization and exploration of inter­molecular close contacts in the crystal structure of the title compound is invaluable. Thus, a Hirshfeld surface (HS) analysis (Hirshfeld, 1977 ▸; Spackman & Jayatilaka, 2009 ▸) was carried out by using *CrystalExplorer17.5* (Turner *et al.*, 2017 ▸) to investigate the locations of atom–atom short contacts with the potential to form hydrogen bonds and the qu­anti­tative ratios of these inter­actions as well as those of the π-stacking inter­actions. In the HS plotted over *d*_norm_ (Fig. 4 ▸), the white surface indicates contacts with distances equal to the sum of van der Waals radii, while the red and blue colours indicate distances shorter (in close contact) or longer (distinct contact) than the van der Waals radii, respectively (Venkatesan *et al.*, 2016 ▸). The bright-red spots appearing near O1, O2, N1, N3 and hydrogen atoms H5, H4, H9*B* and H12 indicate their roles as the respective donors and acceptors in the dominant C—H⋯O and C—H⋯N hydrogen bonds; they also appear as blue and red regions corresponding to positive and negative potentials on the HS mapped over electrostatic potential (Spackman *et al.*, 2008 ▸; Jayatilaka *et al.*, 2005 ▸) shown in Fig. 5 ▸. The blue regions indicate positive electrostatic potential (hydrogen-bond donors), while the red regions indicate negative electrostatic potential (hydrogen-bond acceptors).

**Figure 6:**
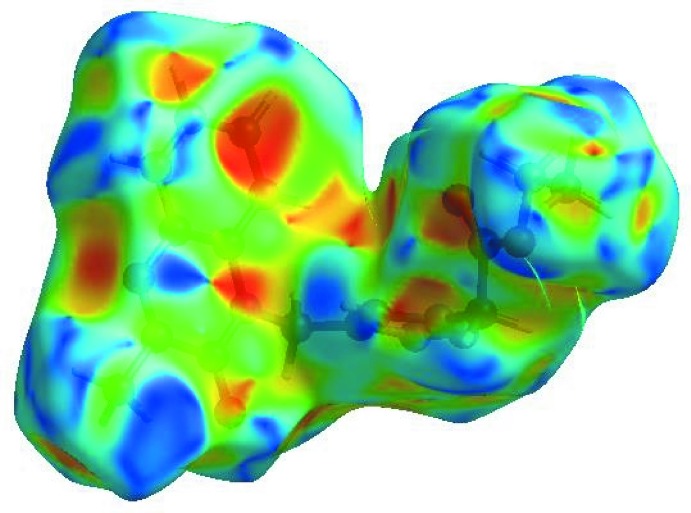
Hirshfeld surface of the title compound plotted over shape-index.

The shape-index of the HS is a tool to visualize π–π stacking inter­actions by the presence of adjacent red and blue triangles; if there are no adjacent red and/or blue triangles, then there are no π–π inter­actions. Fig. 6 ▸ clearly suggest that there are π–π inter­actions present in the title compound.

**Figure 7:**
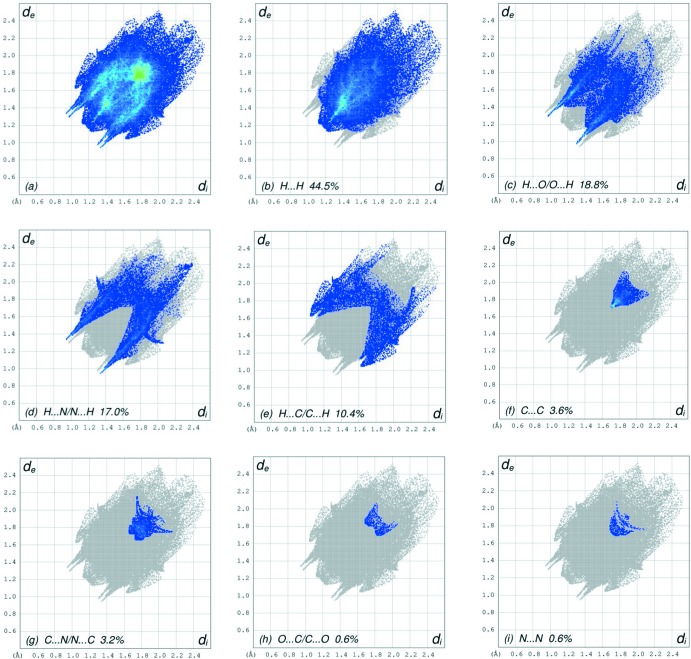
The full two-dimensional fingerprint plots for the title compound, showing (*a*) all inter­actions, and delineated into (*b*) H⋯H, (*c*) H⋯O/O⋯H, (*d*) H⋯N/N⋯H, (*e*) H⋯C/C⋯H, (*f*) C⋯C, (*g*) C⋯N/N⋯C, (*h*) O⋯C/C⋯O and (*i*) N⋯N inter­actions. The *d*_i_ and *d*_e_ values are the closest inter­nal and external distances (in Å) from given points on the Hirshfeld surface contacts.

**Figure 8:**
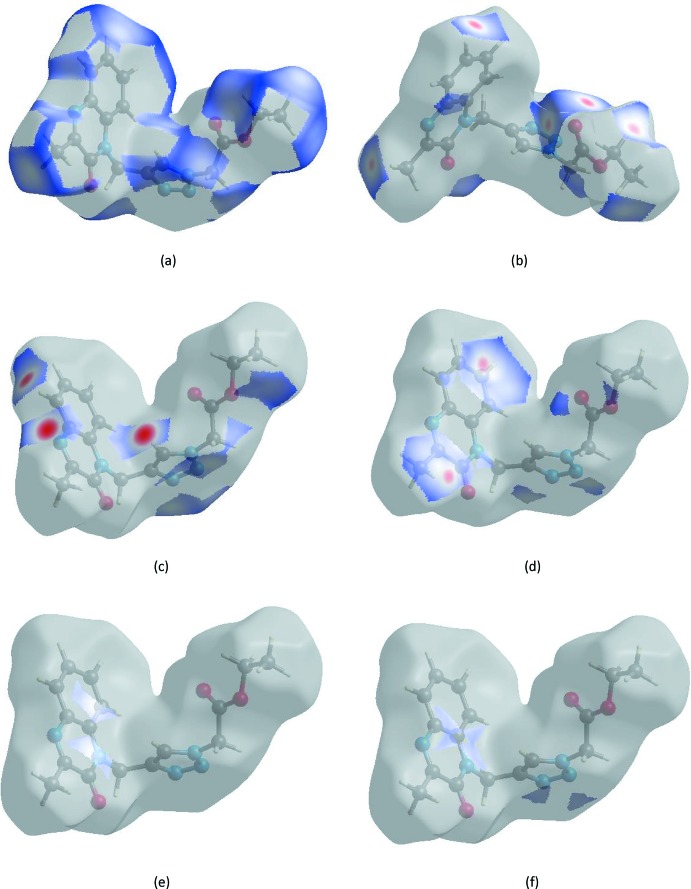
Hirshfeld surface representations with the function *d*_norm_ plotted onto the surface for (*a*) H⋯H, (*b*) H⋯O/O⋯H, (*c*) H⋯N/N⋯H, (*d*) H⋯C/C⋯H, (*e*) C⋯C and (*f*) C⋯N/N⋯C inter­actions.

The overall two-dimensional fingerprint plot is shown in Fig. 7 ▸ *a* and those delineated into H⋯H, H⋯O/O⋯H, H⋯N/N⋯H, H⋯C/C⋯ H, C⋯C, N⋯C/C⋯N, O⋯C/C⋯O and N⋯N contacts (McKinnon *et al.*, 2007 ▸) are illustrated in Fig. 7 ▸ *b*–*i*, respectively, together with their relative contributions to the Hirshfeld surface. The most important inter­action is H⋯H contributing 44.5% to the overall crystal packing, which is reflected in Fig. 7 ▸ *b* as widely scattered points of high density due to the large hydrogen content of the mol­ecule. The wide peak in the centre at *d*_e_ = *d*_i_ = 1.18 Å in Fig. 7 ▸ *b* is due to the short inter­atomic H⋯H contacts (Table 2 ▸). In the fingerprint plot delineated into H⋯O/O⋯H contacts Fig. 7 ▸ *c*, the 18.8% contribution to the HS arises from the inter­molecular C—H⋯O hydrogen bonding (Table 1 ▸) besides the H⋯O/O⋯H contacts (Table 2 ▸) and is viewed as pair of spikes with the tips at *d*_e_ + *d*_i_ ∼ 2.27 Å. The H⋯N/N⋯H contacts in the structure with 17.0% contribution to the HS have a symmetrical distribution of points, Fig. 7 ▸ *d*, with the tips at *d*_e_ + *d*_i_ ∼ 2.30 Å arising from the short inter­atomic C—H⋯N hydrogen bonding (Table 1 ▸) as well as from the H⋯N/N⋯H contacts (Table 3 ▸). The presence of a weak C—H⋯π inter­action (Table 1 ▸) results in two pairs of characteristic wings in the fingerprint plot delineated into H⋯C/C⋯H contacts with a 10.4% contribution to the HS, Fig. 7 ▸ *e*, while the two pairs of thin and thick edges at *d*_e_ + *d*_i_ ∼ 2.77 and 2.67 Å, respectively, result from the inter­atomic H⋯C/C⋯H contacts (Table 2 ▸). The inter­atomic C⋯C contacts (Table 2 ▸) with a 3.6% contribution to the HS appear as an arrow-shaped distribution of points in Fig. 7 ▸ *f*, with the vertex at *d*_e_ = *d*_i_ = 1.71 Å. Finally, the C⋯N/N⋯C (Fig. 7 ▸ *g*) contacts (Table 3 ▸) in the structure, with a 3.2% contribution to the HS, have a symmetrical distribution of points, with a pair of wings appearing at *d*_e_ = *d*_i_ = 1.67 Å. The Hirshfeld surfaces mapped over *d*_norm_ plotted are shown for the H⋯H, H⋯O/O⋯H, H⋯N/N⋯H, H⋯C/C⋯H, C⋯C and C⋯N/N⋯C inter­actions in Fig. 8 ▸ *a*–*f*, respectively.

The Hirshfeld surface analysis confirms the importance of H-atom contacts in establishing the packing. The large number of H⋯H, H⋯O/O⋯H, H⋯ N/N⋯H and H⋯C/C⋯H inter­actions suggest that van der Waals inter­actions and hydrogen bonding play the major roles in the crystal packing (Hathwar *et al.*, 2015 ▸).

## Synthesis and crystallization   

To a solution of 3-methyl-1-(prop-2-yn­yl)-3,4-di­hydro­quinox­alin-2(1*H*)-one (0.65 mmol) in ethanol (20 mL) was added ethyl azido­acetate (1.04 mmol). The mixture was stirred under reflux for 24 h. After completion of the reaction (monitored by TLC), the solution was concentrated and the residue was purified by column chromatography on silica gel by using as eluent a hexa­ne/ethyl acetate (9/1) mixture. Crystals were obtained when the solvent was allowed to evaporate. The solid product isolated was recrystallized from ethanol to afford yellow crystals in 75% yield.

## Refinement   

Crystal data, data collection and structure refinement details are summarized in Table 3 ▸. H atoms were located in a difference-Fourier map and were refined freely. Eleven reflections appearing near the top of the frames on which they were recorded were omitted from the final refinement as they appeared to have been partially obscured by the nozzle of the low-temperature attachment.

## Supplementary Material

Crystal structure: contains datablock(s) global, I. DOI: 10.1107/S2056989018014561/xu5945sup1.cif

Structure factors: contains datablock(s) I. DOI: 10.1107/S2056989018014561/xu5945Isup2.hkl

Supporting information file. DOI: 10.1107/S2056989018014561/xu5945Isup3.cdx

Supporting information file. DOI: 10.1107/S2056989018014561/xu5945Isup4.cml

CCDC reference: 1873385

Additional supporting information:  crystallographic information; 3D view; checkCIF report

## Figures and Tables

**Table 1:** Hydrogen-bond geometry (Å, °) *Cg*3 is the centroid of the benzene (C1–C6) ring.

*D*—H⋯*A*	*D*—H	H⋯*A*	*D*⋯*A*	*D*—H⋯*A*
C5—H5⋯N4^xi^	0.974 (19)	2.48 (2)	3.401 (3)	157.9 (15)
C9—H9*B*⋯O2^iv^	0.97 (2)	2.59 (2)	3.508 (3)	156.9 (18)
C12—H12⋯N1^iv^	0.935 (18)	2.431 (19)	3.365 (2)	177.6 (16)
C13—H13*A*⋯O1^i^	0.99 (2)	2.36 (2)	3.318 (2)	162.5 (16)
C13—H13*B*⋯N3^i^	1.027 (18)	2.672 (19)	3.481 (2)	135.6 (13)
C9—H9*C*⋯*Cg*3^iv^	1.00 (2)	2.67 (2)	3.430 (2)	132.0 (15)

Symmetry codes: (i) 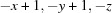; (iv) 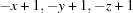; (xi) 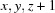.

**Table 2:** Selected interatomic distances (Å)

O1⋯C11	3.394 (3)	N3⋯H13*B* ^i^	2.672 (19)
O1⋯C13^i^	3.318 (3)	N4⋯C5^ix^	3.401 (3)
O1⋯C15^ii^	3.116 (3)	N4⋯H5^ix^	2.48 (2)
O1⋯C16^ii^	3.360 (3)	C1⋯C6^vii^	3.521 (3)
O1⋯H9*A*	2.74 (3)	C1⋯C12	3.519 (3)
O1⋯H10*A*	2.35 (2)	C2⋯C7^vii^	3.459 (3)
O1⋯H13*A* ^i^	2.36 (2)	C2⋯C11	3.397 (3)
O1⋯H15*A* ^ii^	2.61 (2)	C2⋯H10*B*	2.63 (2)
O1⋯H16*A* ^ii^	2.71 (2)	C3⋯C9^vii^	3.574 (3)
O2⋯N5	2.772 (2)	C3⋯H9*A* ^vii^	2.81 (2)
O2⋯C4^iii^	3.409 (3)	C4⋯C8^vii^	3.569 (3)
O2⋯C12	3.186 (2)	C5⋯C8^vii^	3.545 (3)
O2⋯H4^iii^	2.55 (2)	C5⋯C10^vii^	3.548 (3)
O2⋯H9*B* ^iv^	2.59 (2)	C6⋯C7^iv^	3.420 (3)
O2⋯H15*A*	2.72 (2)	C8⋯C12	3.533 (3)
O2⋯H15*B*	2.56 (2)	C10⋯H2	2.61 (2)
O2⋯H16*B* ^v^	2.76 (2)	C11⋯C13^i^	3.421 (3)
O3⋯H15*A* ^vi^	2.84 (3)	C11⋯H2	2.92 (2)
N1⋯N2	2.806 (3)	C11⋯H13*B* ^i^	2.88 (2)
N1⋯C12^iv^	3.365 (3)	C14⋯H16*C* ^vi^	2.95 (2)
N1⋯H12^iv^	2.431 (19)	H2⋯H10*B*	2.17 (2)
N2⋯C6^vii^	3.389 (3)	H3⋯H9*A* ^x^	2.51 (2)
N2⋯H12	2.85 (2)	H10*B*⋯H13*B* ^v^	2.45 (3)
N3⋯H10*A* ^viii^	2.73 (2)		

Symmetry codes: (i) 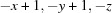; (ii) 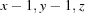; (iii) 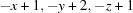; (iv) 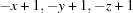; (v) 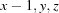; (vi) 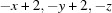; (vii) 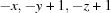; (viii) 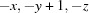; (ix) 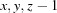; (x) 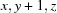.

**Table 3:** Experimental details

Crystal data	
Chemical formula	C_16_H_17_N_5_O_3_
*M*_r_	327.34
Crystal system, space group	Triclinic, *P* 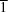
Temperature (K)	100
*a*, *b*, *c* (Å)	7.2061 (15), 10.237 (2), 10.694 (2)
α, β, γ (°)	95.356 (3), 92.867 (3), 100.291 (3)
*V* (Å^3^)	771.0 (3)
*Z*	2
Radiation type	Mo *K*α
μ (mm^−1^)	0.10
Crystal size (mm)	0.25 × 0.24 × 0.13

Data collection	
Diffractometer	Bruker SMART APEX CCD
Absorption correction	Multi-scan (*TWINABS*; Sheldrick, 2009 ▸)
*T*_min_, *T*_max_	0.97, 0.99
No. of measured, independent and observed [*I* > 2σ(*I*)] reflections	14566, 14566, 7794
*R*_int_	0.026
(sin θ/λ)_max_ (Å^−1^)	0.686

Refinement	
*R*[*F* ^2^ > 2σ(*F* ^2^)], *wR*(*F* ^2^), *S*	0.047, 0.151, 1.01
No. of reflections	14566
No. of parameters	286
H-atom treatment	All H-atom parameters refined
Δρ_max_, Δρ_min_ (e Å^−3^)	0.90, −0.53

Computer programs: *APEX3* and *SAINT* (Bruker, 2016 ▸), *SHELXT* (Sheldrick, 2015*a* ▸), *SHELXL2018/1* (Sheldrick, 2015*b* ▸), *DIAMOND* (Brandenburg & Putz, 2012 ▸) and *SHELXTL* (Sheldrick, 2008 ▸).

## supplementary crystallographic information

### Crystal data

**Table:**

C_16_H_17_N_5_O_3_	*Z* = 2
*M**_r_* = 327.34	*F*(000) = 344
Triclinic, *P*1	*D*_x_ = 1.410 Mg m^−^^3^
*a* = 7.2061 (15) Å	Mo *K*α radiation, λ = 0.71073 Å
*b* = 10.237 (2) Å	Cell parameters from 4358 reflections
*c* = 10.694 (2) Å	θ = 2.7–29.1°
α = 95.356 (3)°	µ = 0.10 mm^−^^1^
β = 92.867 (3)°	*T* = 100 K
γ = 100.291 (3)°	Block, gold
*V* = 771.0 (3) Å^3^	0.25 × 0.24 × 0.13 mm

### Data collection

**Table:**

Bruker SMART APEX CCD diffractometer	14566 independent reflections
Radiation source: fine-focus sealed tube	7794 reflections with *I* > 2σ(*I*)
Graphite monochromator	*R*_int_ = 0.026
Detector resolution: 8.3333 pixels mm^-1^	θ_max_ = 29.2°, θ_min_ = 1.9°
ω scans	*h* = −9→9
Absorption correction: multi-scan (*TWINABS*; Sheldrick, 2009)	*k* = −14→13
*T*_min_ = 0.97, *T*_max_ = 0.99	*l* = −14→14
14566 measured reflections	

### Refinement

**Table:**

Refinement on *F*^2^	Primary atom site location: structure-invariant direct methods
Least-squares matrix: full	Secondary atom site location: difference Fourier map
*R*[*F*^2^ > 2σ(*F*^2^)] = 0.047	Hydrogen site location: difference Fourier map
*wR*(*F*^2^) = 0.151	All H-atom parameters refined
*S* = 1.01	*w* = 1/[σ^2^(*F*_o_^2^) + (0.0726*P*)^2^] where *P* = (*F*_o_^2^ + 2*F*_c_^2^)/3
14566 reflections	(Δ/σ)_max_ < 0.001
286 parameters	Δρ_max_ = 0.90 e Å^−^^3^
0 restraints	Δρ_min_ = −0.53 e Å^−^^3^

### Special details

**Table:**

Experimental. The diffraction data were collected in three sets of 363 frames (0.5° width in ω) at φ = 0, 120 and 240°. A scan time of 40 sec/frame was used. Analysis of 226 reflections having I/σ(I) > 12 and chosen from the full data set with *CELL_NOW* (Sheldrick, 2008) showed the crystal to belong to the triclinic system and to be twinned by a 176° rotation about the real axis 1,-0.8,-0.11. The raw data were processed using the multi-component version of *SAINT* under control of the two-component orientation file generated by *CELL_NOW*.
Geometry. All esds (except the esd in the dihedral angle between two l.s. planes) are estimated using the full covariance matrix. The cell esds are taken into account individually in the estimation of esds in distances, angles and torsion angles; correlations between esds in cell parameters are only used when they are defined by crystal symmetry. An approximate (isotropic) treatment of cell esds is used for estimating esds involving l.s. planes.
Refinement. Refinement of F^2^ against ALL reflections. The weighted R-factor wR and goodness of fit S are based on F^2^, conventional R-factors R are based on F, with F set to zero for negative F^2^. The threshold expression of F^2^ > 2sigma(F^2^) is used only for calculating R-factors(gt) etc. and is not relevant to the choice of reflections for refinement. R-factors based on F^2^ are statistically about twice as large as those based on F, and R- factors based on ALL data will be even larger. Refined as a 2-component twin.

### Fractional atomic coordinates and isotropic or equivalent isotropic displacement parameters (Å^2^)

**Table:**

	*x*	*y*	*z*	*U*_iso_*/*U*_eq_	
O1	0.21097 (18)	0.29218 (12)	0.26556 (12)	0.0221 (3)	
O2	0.72802 (18)	0.89513 (13)	0.19527 (13)	0.0253 (3)	
O3	0.99442 (17)	0.86498 (12)	0.10490 (12)	0.0236 (3)	
N1	0.2861 (2)	0.44465 (14)	0.58072 (14)	0.0167 (3)	
N2	0.1816 (2)	0.50319 (14)	0.33885 (14)	0.0149 (3)	
N3	0.2293 (2)	0.62469 (15)	0.02167 (14)	0.0190 (4)	
N4	0.3912 (2)	0.67146 (15)	−0.02467 (14)	0.0195 (4)	
N5	0.5294 (2)	0.66253 (14)	0.06255 (13)	0.0163 (3)	
C1	0.2076 (2)	0.60425 (17)	0.43915 (16)	0.0149 (4)	
C2	0.1859 (3)	0.73515 (18)	0.42255 (19)	0.0197 (4)	
H2	0.156 (3)	0.760 (2)	0.3418 (19)	0.026 (6)*	
C3	0.2093 (3)	0.82977 (19)	0.5254 (2)	0.0232 (4)	
H3	0.198 (3)	0.919 (2)	0.5132 (18)	0.027 (5)*	
C4	0.2543 (3)	0.79803 (19)	0.64554 (19)	0.0221 (4)	
H4	0.266 (3)	0.864 (2)	0.7164 (19)	0.025 (5)*	
C5	0.2805 (3)	0.67017 (19)	0.66222 (18)	0.0193 (4)	
H5	0.317 (3)	0.6456 (19)	0.7445 (18)	0.021 (5)*	
C6	0.2587 (2)	0.57244 (17)	0.55919 (17)	0.0156 (4)	
C7	0.2689 (2)	0.35448 (17)	0.48562 (17)	0.0154 (4)	
C8	0.2200 (2)	0.37824 (17)	0.35441 (17)	0.0156 (4)	
C9	0.2950 (3)	0.21591 (19)	0.5043 (2)	0.0219 (4)	
H9A	0.175 (3)	0.153 (2)	0.472 (2)	0.041 (6)*	
H9B	0.320 (3)	0.207 (2)	0.593 (2)	0.038 (6)*	
H9C	0.401 (3)	0.190 (2)	0.455 (2)	0.039 (6)*	
C10	0.1103 (3)	0.52580 (19)	0.21316 (17)	0.0177 (4)	
H10A	0.045 (3)	0.439 (2)	0.1700 (18)	0.024 (5)*	
H10B	0.014 (3)	0.5837 (18)	0.2229 (17)	0.018 (5)*	
C11	0.2664 (2)	0.58684 (16)	0.13786 (16)	0.0158 (4)	
C12	0.4569 (3)	0.61031 (17)	0.16447 (17)	0.0176 (4)	
H12	0.531 (3)	0.5937 (18)	0.2336 (18)	0.019 (5)*	
C13	0.7253 (3)	0.70767 (18)	0.03953 (18)	0.0179 (4)	
H13A	0.733 (3)	0.7257 (19)	−0.0493 (19)	0.023 (5)*	
H13B	0.803 (2)	0.6349 (19)	0.0549 (17)	0.017 (5)*	
C14	0.8116 (3)	0.83408 (17)	0.12354 (17)	0.0167 (4)	
C15	1.0986 (3)	0.98897 (18)	0.1738 (2)	0.0227 (4)	
H15A	1.045 (3)	1.065 (2)	0.1445 (18)	0.023 (5)*	
H15B	1.075 (3)	0.9888 (19)	0.2649 (19)	0.021 (5)*	
C16	1.3022 (3)	0.9965 (2)	0.1468 (2)	0.0294 (5)	
H16A	1.372 (3)	1.081 (3)	0.191 (2)	0.050 (7)*	
H16B	1.354 (3)	0.922 (2)	0.1786 (19)	0.035 (6)*	
H16C	1.322 (3)	0.992 (2)	0.053 (2)	0.041 (7)*	

### Atomic displacement parameters (Å^2^)

**Table:**

	*U*^11^	*U*^22^	*U*^33^	*U*^12^	*U*^13^	*U*^23^
O1	0.0273 (8)	0.0180 (7)	0.0193 (7)	0.0028 (5)	0.0020 (6)	−0.0040 (5)
O2	0.0247 (7)	0.0203 (7)	0.0289 (8)	0.0022 (6)	0.0064 (6)	−0.0064 (6)
O3	0.0190 (7)	0.0187 (7)	0.0296 (8)	−0.0013 (5)	0.0034 (6)	−0.0070 (6)
N1	0.0147 (8)	0.0181 (8)	0.0175 (8)	0.0030 (6)	0.0013 (6)	0.0029 (6)
N2	0.0155 (8)	0.0154 (7)	0.0133 (8)	0.0015 (6)	−0.0005 (6)	0.0017 (6)
N3	0.0214 (9)	0.0182 (8)	0.0162 (8)	0.0012 (6)	−0.0011 (6)	0.0017 (6)
N4	0.0222 (9)	0.0193 (8)	0.0157 (8)	0.0009 (6)	−0.0021 (6)	0.0020 (6)
N5	0.0188 (8)	0.0148 (7)	0.0137 (8)	0.0002 (6)	−0.0009 (6)	−0.0002 (6)
C1	0.0116 (9)	0.0159 (9)	0.0161 (9)	0.0008 (7)	0.0009 (7)	−0.0006 (7)
C2	0.0179 (10)	0.0178 (9)	0.0235 (11)	0.0032 (7)	−0.0003 (8)	0.0044 (8)
C3	0.0181 (10)	0.0146 (9)	0.0366 (12)	0.0036 (7)	0.0019 (8)	−0.0006 (8)
C4	0.0173 (10)	0.0194 (10)	0.0267 (11)	0.0008 (7)	0.0031 (8)	−0.0085 (8)
C5	0.0145 (9)	0.0233 (10)	0.0182 (10)	0.0000 (7)	0.0023 (8)	−0.0016 (8)
C6	0.0121 (9)	0.0161 (9)	0.0179 (9)	0.0013 (7)	0.0015 (7)	0.0005 (7)
C7	0.0119 (9)	0.0153 (9)	0.0190 (10)	0.0017 (7)	0.0028 (7)	0.0029 (7)
C8	0.0134 (9)	0.0148 (9)	0.0179 (10)	0.0004 (7)	0.0022 (7)	0.0013 (7)
C9	0.0231 (11)	0.0178 (10)	0.0255 (12)	0.0045 (8)	0.0026 (9)	0.0045 (8)
C10	0.0168 (10)	0.0203 (9)	0.0151 (9)	0.0020 (7)	−0.0035 (7)	0.0016 (7)
C11	0.0213 (10)	0.0124 (8)	0.0127 (9)	0.0027 (7)	−0.0017 (7)	−0.0009 (7)
C12	0.0221 (10)	0.0158 (9)	0.0142 (9)	0.0026 (7)	−0.0013 (8)	0.0014 (7)
C13	0.0192 (10)	0.0170 (9)	0.0163 (10)	0.0011 (7)	0.0017 (8)	−0.0004 (7)
C14	0.0201 (10)	0.0137 (8)	0.0164 (9)	0.0028 (7)	0.0007 (7)	0.0025 (7)
C15	0.0231 (11)	0.0156 (9)	0.0262 (12)	−0.0012 (8)	−0.0010 (9)	−0.0046 (8)
C16	0.0220 (11)	0.0218 (11)	0.0419 (14)	0.0008 (8)	−0.0017 (10)	−0.0017 (10)

### Geometric parameters (Å, º)

**Table:**

O1—C8	1.225 (2)	C5—C6	1.400 (2)
O2—C14	1.197 (2)	C5—H5	0.974 (19)
O3—C14	1.328 (2)	C7—C8	1.482 (2)
O3—C15	1.466 (2)	C7—C9	1.494 (2)
N1—C7	1.293 (2)	C9—H9A	1.00 (2)
N1—C6	1.396 (2)	C9—H9B	0.97 (2)
N2—C8	1.379 (2)	C9—H9C	1.00 (2)
N2—C1	1.400 (2)	C10—C11	1.496 (2)
N2—C10	1.468 (2)	C10—H10A	0.99 (2)
N3—N4	1.318 (2)	C10—H10B	0.992 (18)
N3—C11	1.363 (2)	C11—C12	1.362 (3)
N4—N5	1.3511 (19)	C12—H12	0.935 (18)
N5—C12	1.346 (2)	C13—C14	1.519 (2)
N5—C13	1.446 (2)	C13—H13A	0.99 (2)
C1—C6	1.401 (3)	C13—H13B	1.027 (18)
C1—C2	1.403 (2)	C15—C16	1.500 (3)
C2—C3	1.378 (3)	C15—H15A	0.997 (19)
C2—H2	0.95 (2)	C15—H15B	0.996 (19)
C3—C4	1.391 (3)	C16—H16A	0.99 (3)
C3—H3	0.95 (2)	C16—H16B	0.99 (2)
C4—C5	1.382 (3)	C16—H16C	1.02 (2)
C4—H4	0.96 (2)		
			
O1···C11	3.394 (3)	N3···H13B^i^	2.672 (19)
O1···C13^i^	3.318 (3)	N4···C5^ix^	3.401 (3)
O1···C15^ii^	3.116 (3)	N4···H5^ix^	2.48 (2)
O1···C16^ii^	3.360 (3)	C1···C6^vii^	3.521 (3)
O1···H9A	2.74 (3)	C1···C12	3.519 (3)
O1···H10A	2.35 (2)	C2···C7^vii^	3.459 (3)
O1···H13A^i^	2.36 (2)	C2···C11	3.397 (3)
O1···H15A^ii^	2.61 (2)	C2···H10B	2.63 (2)
O1···H16A^ii^	2.71 (2)	C3···C9^vii^	3.574 (3)
O2···N5	2.772 (2)	C3···H9A^vii^	2.81 (2)
O2···C4^iii^	3.409 (3)	C4···C8^vii^	3.569 (3)
O2···C12	3.186 (2)	C5···C8^vii^	3.545 (3)
O2···H4^iii^	2.55 (2)	C5···C10^vii^	3.548 (3)
O2···H9B^iv^	2.59 (2)	C6···C7^iv^	3.420 (3)
O2···H15A	2.72 (2)	C8···C12	3.533 (3)
O2···H15B	2.56 (2)	C10···H2	2.61 (2)
O2···H16B^v^	2.76 (2)	C11···C13^i^	3.421 (3)
O3···H15A^vi^	2.84 (3)	C11···H2	2.92 (2)
N1···N2	2.806 (3)	C11···H13B^i^	2.88 (2)
N1···C12^iv^	3.365 (3)	C14···H16C^vi^	2.95 (2)
N1···H12^iv^	2.431 (19)	H2···H10B	2.17 (2)
N2···C6^vii^	3.389 (3)	H3···H9A^x^	2.51 (2)
N2···H12	2.85 (2)	H10B···H13B^v^	2.45 (3)
N3···H10A^viii^	2.73 (2)		
			
C14—O3—C15	116.32 (14)	C7—C9—H9B	111.1 (13)
C7—N1—C6	118.44 (15)	H9C—C9—H9B	109.7 (17)
C8—N2—C1	121.42 (15)	H9A—C9—H9B	108.8 (17)
C8—N2—C10	117.40 (15)	N2—C10—C11	111.65 (14)
C1—N2—C10	121.18 (14)	N2—C10—H10A	107.9 (11)
N4—N3—C11	108.51 (14)	C11—C10—H10A	110.2 (11)
N3—N4—N5	106.77 (14)	N2—C10—H10B	108.6 (10)
C12—N5—N4	111.21 (15)	C11—C10—H10B	110.9 (10)
C12—N5—C13	128.71 (16)	H10A—C10—H10B	107.5 (15)
N4—N5—C13	120.08 (14)	C12—C11—N3	109.00 (16)
N2—C1—C6	118.25 (15)	C12—C11—C10	129.83 (16)
N2—C1—C2	122.11 (16)	N3—C11—C10	121.13 (16)
C6—C1—C2	119.63 (16)	N5—C12—C11	104.50 (16)
C3—C2—C1	119.48 (18)	N5—C12—H12	123.8 (11)
C3—C2—H2	119.1 (12)	C11—C12—H12	131.7 (11)
C1—C2—H2	121.4 (12)	N5—C13—C14	111.72 (15)
C2—C3—C4	121.25 (18)	N5—C13—H13A	108.9 (11)
C2—C3—H3	119.0 (12)	C14—C13—H13A	108.9 (11)
C4—C3—H3	119.7 (12)	N5—C13—H13B	110.3 (10)
C5—C4—C3	119.61 (18)	C14—C13—H13B	108.8 (10)
C5—C4—H4	120.3 (12)	H13A—C13—H13B	108.1 (15)
C3—C4—H4	120.1 (12)	O2—C14—O3	125.75 (16)
C4—C5—C6	120.24 (18)	O2—C14—C13	125.31 (17)
C4—C5—H5	121.7 (11)	O3—C14—C13	108.93 (15)
C6—C5—H5	118.0 (11)	O3—C15—C16	106.61 (16)
N1—C6—C5	118.17 (16)	O3—C15—H15A	108.2 (11)
N1—C6—C1	122.09 (16)	C16—C15—H15A	112.8 (11)
C5—C6—C1	119.73 (17)	O3—C15—H15B	109.3 (11)
N1—C7—C8	123.89 (16)	C16—C15—H15B	113.9 (11)
N1—C7—C9	120.33 (16)	H15A—C15—H15B	106.0 (15)
C8—C7—C9	115.77 (16)	C15—C16—H16A	106.9 (14)
O1—C8—N2	121.94 (16)	C15—C16—H16B	111.6 (12)
O1—C8—C7	122.44 (16)	H16A—C16—H16B	108.6 (19)
N2—C8—C7	115.60 (15)	C15—C16—H16C	112.4 (13)
C7—C9—H9C	111.2 (13)	H16A—C16—H16C	110.6 (19)
C7—C9—H9A	108.0 (13)	H16B—C16—H16C	106.7 (18)
H9C—C9—H9A	107.9 (18)		
			
C11—N3—N4—N5	0.12 (18)	C1—N2—C8—C7	6.6 (2)
N3—N4—N5—C12	0.01 (19)	C10—N2—C8—C7	−172.74 (14)
N3—N4—N5—C13	−179.45 (14)	N1—C7—C8—O1	177.45 (16)
C8—N2—C1—C6	−5.3 (2)	C9—C7—C8—O1	−3.6 (2)
C10—N2—C1—C6	174.01 (15)	N1—C7—C8—N2	−3.7 (3)
C8—N2—C1—C2	174.05 (16)	C9—C7—C8—N2	175.18 (15)
C10—N2—C1—C2	−6.7 (2)	C8—N2—C10—C11	−95.05 (18)
N2—C1—C2—C3	178.61 (16)	C1—N2—C10—C11	85.64 (19)
C6—C1—C2—C3	−2.1 (3)	N4—N3—C11—C12	−0.20 (19)
C1—C2—C3—C4	0.1 (3)	N4—N3—C11—C10	−178.25 (15)
C2—C3—C4—C5	1.5 (3)	N2—C10—C11—C12	7.1 (3)
C3—C4—C5—C6	−1.1 (3)	N2—C10—C11—N3	−175.33 (15)
C7—N1—C6—C5	−179.00 (16)	N4—N5—C12—C11	−0.13 (19)
C7—N1—C6—C1	2.1 (2)	C13—N5—C12—C11	179.27 (16)
C4—C5—C6—N1	−179.82 (16)	N3—C11—C12—N5	0.20 (19)
C4—C5—C6—C1	−0.9 (3)	C10—C11—C12—N5	178.02 (17)
N2—C1—C6—N1	0.7 (3)	C12—N5—C13—C14	−69.5 (2)
C2—C1—C6—N1	−178.63 (15)	N4—N5—C13—C14	109.82 (17)
N2—C1—C6—C5	−178.19 (15)	C15—O3—C14—O2	−3.5 (3)
C2—C1—C6—C5	2.5 (3)	C15—O3—C14—C13	176.85 (15)
C6—N1—C7—C8	−0.5 (3)	N5—C13—C14—O2	−4.2 (3)
C6—N1—C7—C9	−179.40 (15)	N5—C13—C14—O3	175.41 (14)
C1—N2—C8—O1	−174.62 (15)	C14—O3—C15—C16	175.25 (16)
C10—N2—C8—O1	6.1 (2)		

Symmetry codes: (i) −*x*+1, −*y*+1, −*z*; (ii) *x*−1, *y*−1, *z*; (iii) −*x*+1, −*y*+2, −*z*+1; (iv) −*x*+1, −*y*+1, −*z*+1; (v) *x*−1, *y*, *z*; (vi) −*x*+2, −*y*+2, −*z*; (vii) −*x*, −*y*+1, −*z*+1; (viii) −*x*, −*y*+1, −*z*; (ix) *x*, *y*, *z*−1; (x) *x*, *y*+1, *z*.

### Hydrogen-bond geometry (Å, º)

Cg3 is the centroid of the benzene (C1–C6) ring.

**Table:**

*D*—H···*A*	*D*—H	H···*A*	*D*···*A*	*D*—H···*A*
C5—H5···N4^xi^	0.974 (19)	2.48 (2)	3.401 (3)	157.9 (15)
C9—H9*B*···O2^iv^	0.97 (2)	2.59 (2)	3.508 (3)	156.9 (18)
C12—H12···N1^iv^	0.935 (18)	2.431 (19)	3.365 (2)	177.6 (16)
C13—H13*A*···O1^i^	0.99 (2)	2.36 (2)	3.318 (2)	162.5 (16)
C13—H13*B*···N3^i^	1.027 (18)	2.672 (19)	3.481 (2)	135.6 (13)
C9—H9*C*···*Cg*3^iv^	1.00 (2)	2.67 (2)	3.430 (2)	132.0 (15)

Symmetry codes: (i) −*x*+1, −*y*+1, −*z*; (iv) −*x*+1, −*y*+1, −*z*+1; (xi) *x*, *y*, *z*+1.
